# Incidence and risk factors of postoperative pneumonia following cancer surgery in adult patients with selected solid cancer: results of “Cancer POP” study

**DOI:** 10.1002/cam4.1259

**Published:** 2017-12-22

**Authors:** Jiwon Jung, Song Mi Moon, Hee‐Chang Jang, Cheol‐In Kang, Jae‐Bum Jun, Yong Kyun Cho, Seung‐Ji Kang, Bo‐Jeong Seo, Young‐Joo Kim, Seong‐Beom Park, Juneyoung Lee, Chang Sik Yu, Sung‐Han Kim

**Affiliations:** ^1^ Division of Infectious Diseases Department of Internal Medicine Ulsan University Hospital University of Ulsan College of Medicine Ulsan Korea; ^2^ Department of Infectious Diseases Gachon University Gil Medical Center Incheon Korea; ^3^ Division of Infectious Diseases Department of Internal Medicine Armed Forces Capital Hospital Seongnam Korea; ^4^ Department of Infectious Diseases Chonnam National University Medical School Gwangju Korea; ^5^ Division of Infectious Diseases Department of Internal Medicine Samsung Medical Center Sungkyunkwan University School of Medicine Seoul Korea; ^6^ Outcomes Research/Real World Data Corporate Affairs & Health and Value Pfizer Pharmaceuticals Korea Ltd. Seoul Korea; ^7^ Medical& Scientific Affairs Pfizer Pharmaceuticals Korea Ltd. Seoul Korea; ^8^ Department of Biostatistics College of Medicine Korea University Seoul Korea; ^9^ Department of Colon and Rectal Surgery Asan Medical Center University of Ulsan College of Medicine Seoul Korea; ^10^ Department of Infectious Diseases Asan Medical Center University of Ulsan College of Medicine Seoul Korea

**Keywords:** Breast cancer, colorectal cancer, gastric cancer, hepatocellular carcinoma, lung cancer, postoperative pneumonia

## Abstract

The aim of this study was to investigate the incidence and risk factors of postoperative pneumonia (POP) within 1 year after cancer surgery in patients with the five most common cancers (gastric, colorectal, lung, breast cancer, and hepatocellular carcinoma [HCC]) in South Korea. This was a multicenter and retrospective cohort study performed at five nationwide cancer centers. The number of cancer patients in each center was allocated by the proportion of cancer surgery. Adult patients were randomly selected according to the allocated number, among those who underwent cancer surgery from January to December 2014 within 6 months after diagnosis of cancer. One‐year cumulative incidence of POP was estimated using Kaplan–Meier analysis. An univariable Cox's proportional hazard regression analysis was performed to identify risk factors for POP development. As a multivariable analysis, confounders were adjusted using multiple Cox's PH regression model. Among the total 2000 patients, the numbers of patients with gastric cancer, colorectal cancer, lung cancer, breast cancer, and HCC were 497 (25%), 525 (26%), 277 (14%), 552 (28%), and 149 (7%), respectively. Overall, the 1‐year cumulative incidence of POP was 2.0% (95% CI, 1.4–2.6). The 1‐year cumulative incidences in each cancer were as follows: lung 8.0%, gastric 1.8%, colorectal 1.0%, HCC 0.7%, and breast 0.4%. In multivariable analysis, older age, higher Charlson comorbidity index (CCI) score, ulcer disease, history of pneumonia, and smoking were related with POP development. In conclusions, the 1‐year cumulative incidence of POP in the five most common cancers was 2%. Older age, higher CCI scores, smoker, ulcer disease, and previous pneumonia history increased the risk of POP development in cancer patients.

## Introduction

The incidence of pneumonia in cancer patients is high due to impaired immune function caused by cancer itself and chemotherapy 1. In addition, surgery, a treatment modality for cancer, can cause additional lung injury 2 which may make cancer patients be more susceptible to the development of pneumonia. Therefore, many authorities recommend pneumococcal and influenza vaccination in cancer patients 3. However, these recommendations are not based on clinical data regarding the incidence of pneumonia in various cancer patients and the specific type of pneumonia. Actually, there are limited data on the incidence of postoperative pneumonia (POP) following major cancer surgery. The incidence of POP after lung cancer surgery ranged from 2.1% to 10.7% 4, 5, 6, 7, 8, 9, 10, 11, after gastric cancer surgery 3.6% to 4.3% 12, 13, and after colorectal cancer surgery 6.2% 14. To our knowledge, however, there have been few studies conducted on the incidence of POP after surgery in other cancers or comparative analyses of the incidence of various cancer types. Therefore, a comparison of the incidence of POP in various common cancer patients and the identification of risk factors for POP provides important information to build vaccine priority and other preventive strategies. Therefore, our aim was to determine the incidence and risk factors of POP within 1 year after cancer surgery in patients with the five most common cancers (gastric, colorectal, lung, breast cancer, and hepatocellular carcinoma [HCC]) at five nationwide cancer centers in South Korea.

## Methods

### Study design and population

We conducted a retrospective observational study at five nationwide cancer centers (Asan Medical Center, a 2700‐bed tertiary referral center located in Seoul; Samsung Medical Center, a 1950‐bed tertiary referral center in Seoul; Gachon University Gil Medical Center, a 1400‐bed tertiary referral center in Incheon; Ulsan University Hospital, 940‐bed tertiary referral center in Ulsan; and Hwasun Chonnam National University Hospital, a 700‐bed tertiary referral center in Gwangju in South Korea). Cancer and subsequent cancer surgery were considered exposure factors. We aimed to determine the overall incidence of POP in cancer patients who met the following inclusion criteria of this study: (1) adults (≥19 years old); (2) principal diagnosis of gastric cancer, colorectal cancer, lung cancer, breast cancer, or HCC; and (3) underwent cancer surgery from January 2014 to December 2014, within 6 months after cancer diagnosis. Cancer patients who participated in other clinical trials were excluded from this study because the administration of new chemotherapy or immunomodulating agents may affect the immunity and incidence of POP. Allocations of the number of patients in each center were according to the number of cancer surgeries performed in each center. The patients were randomly selected according to the allocated number of each center. The site investigators gathered the data from the medical records of patients from April to August 2016. The study protocols were approved by the independent institutional review board at each study site.

### Definition

Diagnosis of pneumonia was established based on the ICD‐10 diagnostic code at a hospital discharge record and confirmed by the investigator upon identifying the presence of a new or progressive infiltrate on chest radiography or computed tomography, together with any of the following: new onset of purulent sputum, change in character of chronic sputum, fever ≥38°C, a new rise in C‐reactive protein value or WBC count, positive blood cultures, or isolation of pathogen from sputum, transtracheal aspirate or bronchial washing. Community‐acquired pneumonia was defined as pneumonia that occurs <48 h after admission if the patient did not have contact with health care, and hospital‐acquired pneumonia (HAP) is defined as pneumonia that occurs 48 h or more after admission. Healthcare‐associated pneumonia (HCAP) is defined as pneumonia in patients who were hospitalized in an acute care hospital for two or more days within 90 days of the infection; resided in a nursing home or long‐term care facility; received recent intravenous antibiotic therapy, chemotherapy, or wound care within the past 30 days of the current infection; or attended a hospital or hemodialysis clinic 15.

### Data collection

Demographic characteristics (age, sex, body mass index, and smoking history), comorbidity, history of previous surgery or pneumonia, results of pulmonary function test before surgery, previous vaccinations, type and stage of cancer, receipt of neoadjuvant or adjuvant therapy, date of diagnosis of cancer and surgery, duration of anesthesia and surgery, the amount of bleeding during surgery, and development of POP were retrospectively collected from the medical records. A bacterial pathogen was determined to be present if Gram‐positive or Gram‐negative bacteria were detected in blood, sputum, endotracheal aspirate, bronchoalveolar lavage specimen by culture or PCR assay; if *Chlamydia pneumoniae* or *Mycoplasma pneumoniae* was detected in a nasopharyngeal or oropharyngeal swab by means of PCR assay; if *Legionella pneumophila* was detected in sputum by means of PCR assay; or if *L. pneumophila* or *Streptococcus pneumoniae* was detected in urine by means of antigen detection^16^. A viral pathogen was determined to be present if adenovirus, coronavirus, human metapneumovirus, human rhinovirus, influenza virus, parainfluenza virus, or respiratory syncytial virus was detected in a nasopharyngeal or oropharyngeal swab by means of PCR assay 16.

### Statistical analysis

According to the annual report of the Korea Central Cancer Registry in 2002, 31,808 patients underwent cancer surgery due to solid cancer in 2002 17. A previous study showed that the incidence of POP after operation for lung cancer was 3.6% 8. A total of 2000 patients was, therefore, projected to estimate the POP incidence with an error margin of ±0.8% using a two‐sided 95% confidence interval with the finite population of approximately 31,800 patients 18. The number of patients recruited was proportionately assigned to each of the hospitals according to their cancer surgery volume. Demographic and clinical characteristics of study subjects were summarized as mean (SD) or number of subjects (its percentage). One‐year cumulative incidence of POP was estimated using Kaplan–Meier analysis. An univariate Cox's proportional hazard (PH) regression analysis was performed to identify potential risk factors for POP incidence in cancer patients. Variables with *P* < 0.05 were included in multiple Cox's PH regression model to identify independent risk factors associated with the incidence. The development of POP in patients with lung cancer may be associated with lung cancer surgery itself, and we assumed that the risk factors for POP in patients with other cancers may be different with that in patients with lung cancer. Therefore, we performed multivariable analyses to identify the risk factors in patients with lung cancer and non‐lung cancer, separately. Four multivariable models (using all patients with and without a variable of cancer type, using lung cancer patients only, using cancer patients other than lung cancer) were examined.

All statistical analyses were performed using the SAS software, version 9.4 (SAS Institute Inc., Cary, NC), and variables having a two‐sided *P *< 0.05 were considered statistically significant.

## Results

### Patient characteristics

Of the 2000 patients, excluding 118 patients who were enrolled in other clinical trials, those with gastric cancer, colorectal cancer, lung cancer, breast cancer, and HCC were 497 (25%), 525 (26%), 277 (14%), 552 (28%), and 149 (7%), respectively. The mean ± SD age was 59 ± 12 years, and 48% of all patients were male (Table 1). The number of participants in the ever‐smoker group was 719 (36%), and the most common underlying disease was diabetes mellitus (14%) followed by mild liver disease (7%), and chronic pulmonary disease (5%). The mean ± SD Charlson comorbidity index (CCI) score was 0.4 ± 0.6. About half of all patients (1112 [56%]) received neoadjuvant or adjuvant chemotherapy or radiation therapy.

**Table 1:** Demographic and clinical characteristics of the 2000 patients

Characteristics	Summary statistic
Age (years), mean ± SD	59 ± 12
Old age (≥65 years)	711 (36)
Male	969 (48)
BMI (kg/m^2^), mean ± SD	24 ± 3
Smoking history	719 (36)
Comorbidity^a^	
Diabetes mellitus	280 (14)
Mild liver disease	143 (7)
Chronic pulmonary disease	97 (5)
Metastatic solid tumor	73 (4)
Other solitary tumors	56 (3)
Cerebrovascular disease	54 (3)
Alcoholism	22 (1)
Moderate‐to‐severe renal disease	15 (0.8)
Moderate‐to‐severe liver disease	14 (0.7)
Ulcer disease	14 (0.7)
Dementia	9 (0.5)
Myocardial infarction	8 (0.4)
CCI scores, mean ± SD	0.4 ± 0.6
Pneumonia history	35 (2)
Operation history	975 (49)
Initial pulmonary function test	
FEV_1_ < 80% (*n* = 1601)	304 (19)
FEV_1_/FVC < 70% (*n* = 1600)	25 (2)
DLco < 80% (*n* = 302)	109 (36)
Cancer type	
Breast cancer	552 (28)
Colorectal cancer	525 (26)
Gastric cancer	497 (25)
Lung cancer	277 (14)
Hepatocellular carcinoma	149 (7)
Cancer stage	
Tumor size	
T1	892 (45)
T2 or T3	944 (47)
T4	164 (8)
Nodes involved	
N0	1415 (71)
N1‐N3	585 (29)
Metastasis	
M0	1900 (95)
M1	100 (5)
Neoadjuvant or adjuvant therapy	1112 (56)
Duration of anesthesia (minutes), mean ± SD	206 ± 99
Duration of operation (minutes), mean ± SD	173 ± 90
Surgery	
Video‐assisted (or laparoscopic) surgery	800 (40)
Open surgery	1161 (58)
Conversion from Video‐assisted to open surgery	33 (2)
Amount of bleeding (mL), mean ± SD	172 ± (283)
Received RBC transfusion	52 (3)
Time to resume diet (hours), mean ± SD	51 ± 44
Time to ambulation (hours), mean ± SD	35 ± 37
Incidence of POP	39 (2)
Time to development of POP (weeks), mean ± SD	14 ± 15
Type of POP	
Community‐acquired pneumonia	8 (21)
Healthcare‐associated pneumonia	10 (26)
Hospital‐acquired pneumonia	21 (54)
Ventilator‐associated pneumonia	0 (0)
Pathogen	
*Staphylococcus aureus*	5 (13)
*Streptococcus pneumoniae*	2 (5)
*Acinetobacter baumanii*	2 (5)
*Pseudomonas aeruginosa*	1 (3)
Respiratory virus	4 (10)
Influenza virus	2 (5)
Rhinovirus	1 (3)
Human metapneumovirus	1 (3)
Others	5 (13)
Unidentified	23 (59)

Data are presented as the number (%) of patients unless indicated otherwise. SD, standard deviation; BMI, body mass index; CCI, Charlson comorbidity index; FEV_1_, force expiratory volume in 1 sec; FVC, force vital capacity; DC_LC_, diffusing capacity of the lung for carbon monoxide; RBC, red blood cell; POP, postoperative pneumonia.

a Multiple response item.

### Pneumonia incidence

**Figure 1:**
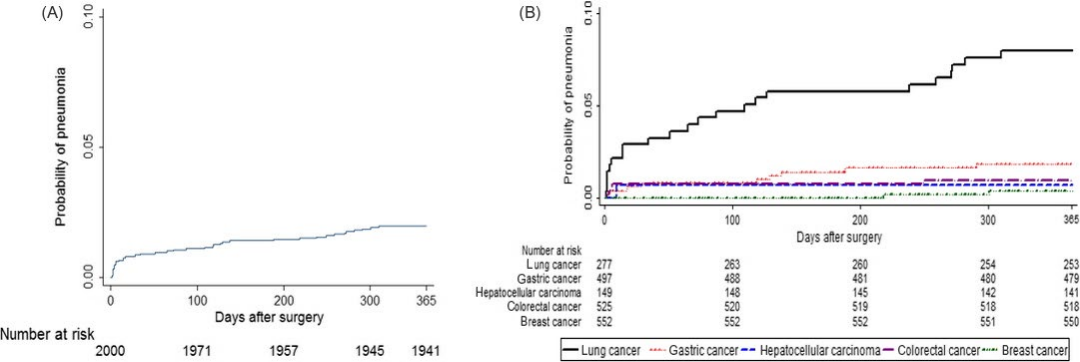
One‐year cumulative incidence of postoperative pneumonia with total patients (A) as well as by cancer type (B) (*N* = 2000).

Thirty‐nine patients developed POP; the overall 1‐year cumulative incidence of POP was 1.96% (95% CI, 1.35–2.57) (Table 2 and Fig. 1A). The 1‐year cumulative incidence in each cancer was as follows: lung 7.97%, gastric 1.82%, colorectal 0.96%, HCC 0.67%, and breast 0.36% (Table 2). Of the 39 cases with POP, 8 (21%) were classified as community‐acquired pneumonia, 10 (26%) were as healthcare‐associated pneumonia, and 21 (54%) were as hospital‐acquired pneumonia. The time to the development of POP after surgery stratified by the classification of pneumonia is shown in Figure S1. The pathogen was identified in 13 of 39 (41%) patients; *Staphylococcus aureus* in five (13%) patients, *S. pneumoniae* in two (5%), *Acinetobacter baumanii* in two (5%), *Pseudomonas aeruginosa* in one (3%), and influenza virus in two (5%) patients (Table 1). Of the 39 patients, 18 were confirmed by chest CT as well. In addition, the overall 30‐day cumulative incidence of POP was 0.85% (95% CI, 0.45–1.25) and those in each cancer were as follows: 2.89% in lung cancer, 0.81% in gastric cancer, 0.76% in colorectal cancer, 0.67% in HCC, and 0% in breast cancer.

**Table 2:** One‐year cumulative incidence of postoperative pneumonia with total patients as well as by cancer type

	No. of patients	No. of POP	Estimated 1‐year cumulative incidence (%) (95% CI)^a^	HR (95% CI)^b^	*P*‐value
Total	2000	39	1.96 (1.35, 2.57)		
Lung cancer	277	22	7.97 (4.78, 11.17)	22.9 (5.38, 97.2)	<0.0001
Gastric cancer	497	9	1.82 (0.64, 3.01)	5.08 (1.10, 23.5)	0.04
Colorectal cancer	525	5	0.96 (0.12, 1.79)	2.65 (0.51, 13.7)	0.61
Hepatocellular carcinoma	149	1	0.67 (0.00, 1.98)	1.88 (0.17, 20.7)	0.24
Breast cancer	552	2	0.36 (0.00, 0.86)	Reference	–

No, number; POP, postoperative pneumonia; CI, confidence interval; HR, hazard ratio.

a By Kaplan–Meier analysis.

b By univariable Cox's PH regression analysis.

As shown in Table 2, the HR (95% CI) for development of POP incidence in patients with lung cancer, gastric cancer, colorectal cancer, and HCC were 22.9 (5.38–97.2) (*P *<* *0.0001), 5.08 (1.10–23.5) (*P *=* *0.04), 2.65 (0.51–13.7) (*P *=* *0.61) and 1.88 (0.17–20.7) (*P *=* *0.24), respectively, compared to those with breast cancer as a referent. The Kaplan–Meier analysis showed that patients with lung cancer developed POP more frequently than patients with other cancers (long‐rank *P *<* *0.001; Fig. 1B). Interestingly, the cumulative incidence of POP in patients with gastric cancer was higher than the composite cumulative incidence of POP in patients with colorectal cancer, HCC, and breast cancer after 100 days from the surgery (log‐rank *P* = 0.03; Fig. S2).

### Risk factors for development of POP

In univariable Cox regression analysis, the following variables were associated with POP: male, older age, smoker, higher CCI score, chronic pulmonary disease, ulcer disease, moderate‐to‐severe liver disease, previous pneumonia history, duration of operation, conversion from video‐associated surgery to open surgery, and more bleeding during the surgery (Table 3).

**Table 3:** One‐year cumulative incidence of postoperative pneumonia according to patient's characteristics (*N* = 2000)

	No. of patients	No. of POP	Estimated 1‐year cumulative incidence (%) (95% CI)^a^	HR (95% CI)^b^	*P*‐value
Gender					
Male	969	31	3.21 (2.10, 4.33)	4.19 (1.93, 9.11)	0.0003
Female	1031	8	0.78 (0.24, 1.31)	Ref	–
Age					
≥65 years	711	26	3.68 (2.29, 5.07)	3.70 (1.90, 7.19)	0.0001
< 65 years	1289	13	1.01 (0.46, 1.56)	Ref	–
BMI (kg/m^2^)	1997	39		0.96 (0.87, 1.06)	0.46
Smoking history					
Ever smoker	719	29	4.05 (2.61, 5.5)	5.2 (2.53, 10.7)	<0.0001
Never smoker	1264	10	0.79 (0.30, 1.28)	Ref	–
CCI scores	2000	39		2.91 (1.96, 4.33)	<0.0001
Chronic pulmonary disease					
Yes	97	8	8.27 (2.78, 13.76)	5.22 (2.40, 11.4)	<0.0001
No	1903	31	1.64 (1.06, 2.21)	Ref	–
Ulcer disease					
Yes	14	2	14.29 (0.00, 32.62)	8.41 (2.03, 34.9)	0.003
No	1986	37	1.87 (1.27, 2.47)	Ref	–
Moderate‐to‐severe liver disease					
Yes	14	2	14.29 (0.00, 32.62)	8.03 (1.94, 33.3)	0.004
No	1986	37	1.87 (1.27, 2.47)	Ref	–
Pneumonia history					
Yes	35	4	11.61 (0.91, 22.31)	6.50 (2.31, 18.3)	0.004
No	1883	34	1.81 (1.21, 2.41)	Ref	–
Neoadjuvant or adjuvant therapy					
Yes	1112	23	2.08 (1.24, 2.92)	1.1 (0.6, 2.2)	0.68
No	888	16	1.81 (0.93, 2.68)	Ref	–
Duration of the operation (min)	2000	39		1.003 (1.0, 1.005)	0.003
Duration of the anesthesia (min)	1996	39		1.002 (1.0, 1.005)	0.06
Open surgery	1161	22	1.91 (1.12, 2.69)	1.09 (0.56, 2.12)	0.81
Conversions	33	3	9.09 (0.0, 18.9)	5.38 (1.55, 18.7)	0.01
Video‐assisted operation	800	14	1.75 (0.84, 2.66)	Ref	–
Amount of bleeding (ml)	1572	30		1.001 (1.0, 1.001)	0.01
Time to resume diet (hours)	1937	39		0.99 (0.98, 1.0)	0.08
Time to ambulation (hours)	1027	24		1.01 (1.0, 1.01)	0.09

No, number; POP, postoperative pneumonia; CI, confidence interval; HR, hazard ratio; Ref, reference; OP, operation; min, minutes; CCI, Charlson comorbidity index.

a By Kaplan–Meier analysis.

b By univariable Cox's PH regression analysis.

Table 4 demonstrates the multiple analysis of risk factors for the development of POP. The significant variables in the univariable analysis were included in a multiple Cox's PH regression model 1; this model indicated that older age, smoker, higher scores of CCI, ulcer disease, and pneumonia history were independent risk factors for development of POP among all patients. Model 2 included significant variables in the univariable analysis and cancer type as an independent variable; higher score of CCI, moderate‐to‐severe liver disease, and lung cancer were independent risk factors for development of POP. In addition, in patients with lung cancer, smoker, higher scores of CCI, and moderate‐to‐severe liver disease were associated with the development of POP (model 3). Finally, in patients with other cancers, chronic pulmonary disease was associated with the development of POP (model 4).

**Table 4:** Risk factors for an incidence of postoperative pneumonia among patients with cancer

	Model 1	Model 2	Model 3	Model 4
	HR (95% CI)^a^	HR (95% CI)^a^	HR (95% CI)^a^	HR (95% CI)^a^
Male (Ref: female)	0.814 (0.265, 2.506)	1.084 (0.314, 3.74)	0.453 (0.071, 2.903)	1.355 (0.295, 6.221)
Old age (≥65 years) (Ref: <65)	3.158 (1.567, 6.364)^b^	2.031 (0.988, 4.175)	1.086 (0.417, 2.827)	2.903 (0.963, 8.745)
Smoking history	4.627 (1.661, 12.892)^b^	2.43 (0.789, 7.481)	10.141 (1.127, 91.224)^b^	1.796 (0.47, 6.738)
CCI score	2.035 (1.242, 3.333)^b^	2.265 (1.392, 3.683)^b^	2.879 (1.527, 5.429)^b^	1.294 (0.549, 3.052)
Chronic pulmonary disease	1.153 (0.474, 2.803)	1.133 (0.469, 2.74)	0.351 (0.078, 1.587)	5.011 (1.439, 17.454)^b^
Ulcer disease	6.244 (1.407, 27.713)^b^	3.977 (0.911, 17.368)	4.989 (0.643, 38.698)	6.594 (0.645, 67.404)
Moderate‐to‐severe liver disease	3.67 (0.757, 17.797)	5.049 (1.005, 25.364)^b^	23.561 (1.162, 477.545)^b^	6.313 (0.58, 68.767)
Pneumonia history	3.012 (1.051, 8.629)^b^	1.215 (0.398, 3.708)	0.794 (0.205, 3.08)	
Lung cancer (Ref: breast cancer)		5.431 (1.014, 29.08)^b^		
Gastric cancer (Ref: breast cancer)		1.416 (0.245, 8.186)		
Hepatocellular carcinoma (Ref: breast cancer)		0.3 (0.022, 4.14)		
Colorectal cancer (Ref: breast cancer)		0.789 (0.129, 4.839)		
Duration of the operation (min)	1.001 (0.998, 1.004)	1.002 (0.998, 1.006)	1.006 (0.999, 1.012)	1 (0.995, 1.005)

Model 1: with all patients; Model 2: with all patients without considering cancer type as an independent variable; Model 3: with lung cancer patients only; Model 4: with the other cancer patients. In model 4, variable of pneumonia history was excluded from the model due to its nonconvergence. HR, hazard ratio; CI, confidence interval; Ref, reference; CCI, Charlson comorbidity index; min, minutes.

a By Multivariable Cox's PH regression analysis.

b Variables with *P* < 0.05.

## Discussion

In the present study, we observed that the 1‐year cumulative incidence of POP in the five most common cancers was 2% in South Korea. Of the selected cancers, the incidence of POP was highest in lung cancer. Older age, smoking history, higher CCI score, ulcer disease, and history of previous pneumonia were independent risk factors for development of POP in cancer patients.

The POP incidence in lung cancer in this study (8%) was within the range of that in previous reports (2.1–10.7%) 4, 5, 6, 7, 8, 9, 10, 11. However, the incidences of POP in patients with gastric cancer and colorectal cancer in the present study (1.8% and 0.96%) were lower than those in previous reports (3.6–4.3% for gastric cancer 12, 13 and 6.2% for colorectal cancer 14). The reason for these discrepancies is not clear. The possible explanations may be difference of the characteristics of the study population, perioperative management, and the period of study. Study population in two previous Japanese studies identifying the incidence and risk factors of POP after gastric cancer surgery were older than our patients with gastric cancer (median age 68 years in the Japanese study vs. a mean age of 59 years in this study), and the durations of the operations in the previous studies 12, 13 were longer than those in our study. The study identifying the incidence of POP in patients with colorectal cancer was conducted 20 years ago 14, and the increased proportion of laparoscopic surgery and improved perioperative management may be associated with the declined incidence of POP in colorectal cancer in the present study. Additionally, this is one of the few studies comparing the incidence of POP by cancer type. We have clearly shown that the incidence of POP was highest in lung cancer, followed by gastric cancer, colorectal cancer, HCC, and breast cancer. This finding may provide information on the policy of pneumococcal or influenza vaccination priority in common cancer; for example, patients with lung cancer are vaccinated in priority. However, the number of confirmed cases of pneumococcal and influenza pneumonia was small in this study, so some may argue that our data are insufficient to guide policy considerations. Despite of this, pneumococcal and influenza vaccines in cancer patients have been recommended in clinical practice 3 although there was limited information on the specific type of pneumonia in cancer patients. Therefore, given the limited sensitivity of microbiologic diagnostic methods, our data should be interpreted with great caution to prioritize those patients who need vaccination.

Interestingly, the incidence of POP in patients with gastric cancer was higher than that in patients with colorectal cancer, HCC, and breast cancer at 100 days after cancer surgery (Fig. S2). Aspiration of esophageal reflux contents, malnutrition, and decreased immunity could be responsible for this novel finding 19, 20, 21. Kaneda et al. showed a higher incidence of POP among patients who underwent lung cancer surgery with a history of gastrectomy 19. Similarly, the history of gastrectomy has been considered a risk factor for tuberculosis, suggesting that gastrectomy may affect cell‐mediated immunity 20, 21, 22. Further biologic plausibility for these findings should be addressed in future studies.

Various factors have been found to increase POP in previous studies; male gender 13, older age 5, 6, 7, 8, 10, 11, 12, 23, 24, obesity 8, malnutrition 24, previous pneumonia 8, diabetes mellitus 12, chronic pulmonary disease 8, alcoholism 8, atrial fibrillation 8, CCI score 6, type of surgery 6, 11, 23, 24, duration of surgery 23, pulmonary function test 5, 7, 11, 12, 23, perioperative transfusion 5, 12, 13, postoperative complication other than pneumonia 5, advanced cancer stage 24, and induction therapy 7 were associated with POP development. In the present study, we showed that older age, smoker, higher CCI score, peptic ulcer disease, and history of previous pneumonia were independent risk factors for development of POP among all patients. The association of peptic ulcer disease and POP, although the number of patients with a history of peptic ulcer (*n* = 14) is too small to draw a firm conclusion, may be related to the use of proton‐pump inhibitor, which raises gastric pH resulting in an overgrowth of bacteria. There have been reports that the use of acid‐suppressive medication increased the risk for pneumonia 25, 26, 27. In addition, when the cancer type was included as variables in the multivariable analysis (model 2), we demonstrated that lung cancer itself was an independent risk factor after adjustment for other factors. Therefore, clinicians should be aware of the heightened risk of POP in patients with these risk factors and postoperative care including frequent coughing, deep breathing, early ambulation, and use of a spirometer 28 cannot be overemphasized for preventing POP.

There are limitations of note in this study. First, since the diagnosis of pneumonia was established according to the ICD‐10 diagnostic code on each hospital's discharge record, the incidence of POP may be underreported. Second, the follow‐up period after cancer surgery was 1 year. Some may question that a 1‐year follow‐up period is arbitrary. We assumed that the development of POP is highest during the 1 year after surgery and then gradually becomes lower because surgery and perioperative chemoradiation therapy may affect the occurrence of pneumonia. However, further studies are warranted to obtain long‐term follow‐up data in cancer patients who received surgery. Third, since there were few incidences of POP in patients with HCC and breast cancer, our findings should be cautiously interpreted in these patients. Fourth, of the 30 patients who died during the follow‐up period, a total of 20 patients died without developing POP. As these patients were treated as a censoring event, not as a competing risk, there was a possibility of our estimate of hazard ratio for development of POP being slightly overestimated. Finally, the pathogen identification rate was low, and the incidence of pneumococcal pneumonia may be underreported. However, the pathogen is usually not detected in half of patients with pneumonia in clinical settings 29. In addition, the previous study evaluating the incidence and pathogens of community‐acquired pneumonia requiring hospitalization in adult patients showed that the pathogen was detected in only 38% of patients 16. In this context, the pathogen identification rate (41%) in the present study is in line with previous studies. Therefore, the low proportion of *S. pneumoniae* as an etiologic agent in POP should be cautiously interpreted due to the insensitivity of sputum culture.

In conclusion, the overall 1‐year cumulative incidence of POP was 2% in total patients with selected solid cancers, 8% in patients with lung cancer, and 2% in patients with gastric cancer in South Korea. Older age, higher CCI scores, smoker, ulcer disease, and previous pneumonia history were independent risk factors for the development of POP in cancer patients. Therefore, more cautious monitoring the development of POP in patients with these risk factors would be helpful.

## Conflict of Interest

No author has any conflict of interest regarding this study.

## Supporting information


**Figure S1.** Time to development of POP after surgery stratified by types of pneumonia.
**Figure S2.** POP cumulative incidence in gastric cancer versus the composite POP cumulative incidence of colorectal cancer, hepatocellular carcinoma and breast cancer within 100 days from the surgery (A) and after 100 days from the surgery (B).
