# Supplementary material for: Incidence and risk factors of postoperative pneumonia following cancer surgery in adult patients with selected solid cancer: results of “Cancer POP” study
Source: Cancer Med. 2017 Dec 22;7(1):261–9. doi: 10.1002/cam4.1259 (PMC5773948; doi:10.1002/cam4.1259)

**Supplemental Figure 1. Time to development of POP after surgery stratified by types of pneumonia**

**
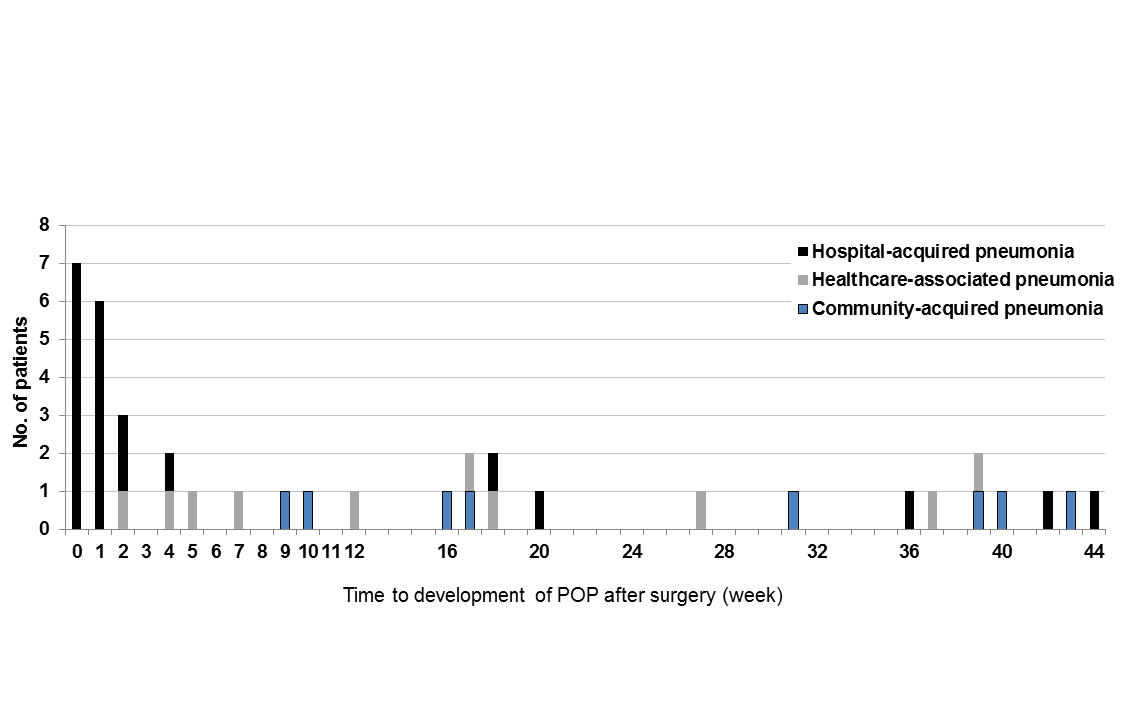
Supplemental Figure 2. POP cumulative incidence in gastric cancer vs. the composite POP cumulative incidence of colorectal cancer, hepatocellular carcinoma and breast cancer within 100 days from the surgery (A) and after 100 days from the surgery (B).**


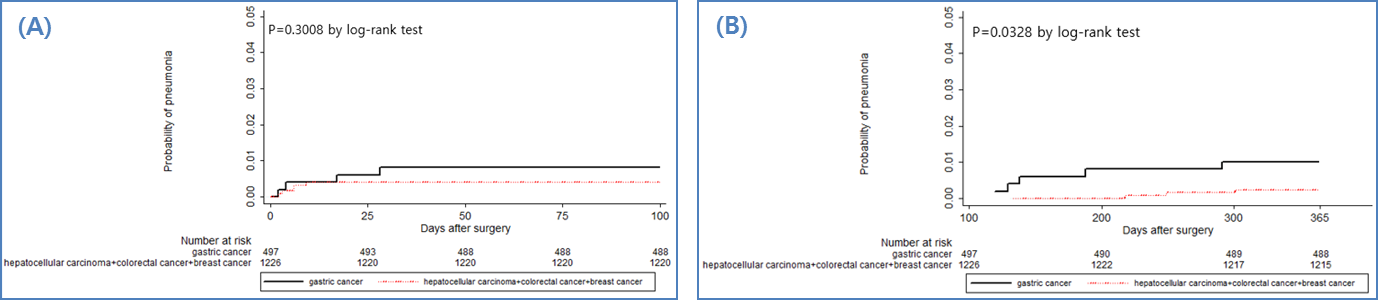

Supplement: Supplementary file 1 — Figure S1. Time to development of POP after surgery stratified by types of pneumonia. Figure S2. POP cumulative incidence in gastric cancer versus the composite POP cumulative incidence of colorectal cancer, hepatocellular carcinoma and breast cancer within 100 days from the surgery (A) and after 100 days from the surgery (B). [file CAM4-7-261-s001.docx]
